# An assessment of the growth inhibition profiles of *Hamamelis virginiana* L. extracts against *Streptococcus* and *Staphylococcus* spp.

**DOI:** 10.1016/j.jtcme.2021.03.002

**Published:** 2021-04-01

**Authors:** Matthew J. Cheesman, Sean Alcorn, Vishal Verma, Ian E. Cock

**Affiliations:** a School of Pharmacy and Pharmacology, Gold Coast Campus, Griffith University, Australia; b Menzies Health Institute Queensland, Quality Use of Medicines Network, Australia; c School of Environment and Science, Nathan Campus, Griffith University, Australia; d Environmental Futures Research Institute, Nathan Campus, Griffith University, Australia

**Keywords:** Witch-hazel, Staphylococcus, Streptococcus, Extracts, Antibacterial

## Abstract

Staphylococcal and streptococcal species trigger a wide variety of infections involving epithelial tissues. Virginian witch hazel (WH; *Hamamelis virginiana* L.; family: Hamamelidaceae) is a plant that has been used traditionally by Native Americans to treat a variety of skin conditions. Extracts from the leaves were examined for their inhibitory effects on these bacterial species. Solvents of different polarity (water, methanol, ethyl acetate, hexane and chloroform) were used to prepare extracts from WH leaves, and the aqueous resuspensions were screened for antibacterial activities using disc diffusion and liquid dilution assays. Extract phytochemical profiles and toxicities were also examined, and combinations of extracts with conventional antibiotics were tested against each bacterial strain. The methanolic and aqueous extracts inhibited the growth of *S. oralis*, *S. pyogenes*, *S. epidermidis* and *S. aureus*, but not *S. mutans*. The extracts were especially active against staphylococcal species, with MIC values between 200 and 500 μg/ml. Combinations of active extracts with conventional antibiotics failed to yield beneficial interactions, except for two cases where additive interactions were observed (aqueous WH extract combined with chloramphenicol against *S. oralis*, and methanolic WH extract combined with ciprofloxacin against *S. aureus*). Phytochemical assays indicated an abundance of tannins, triterpenoids and phenolics in the water and methanol extracts, with trace amounts of these components in the ethyl acetate extract. Phytochemicals were not detected in hexane and chloroform extracts. Thus, phytochemical abundance in extracts was concordant with antibacterial activities. All extracts were found to be non-toxic in *Artemia nauplii* assays. These findings indicate the potential for WH leaf extracts for clinical use in treating staphylococcal and streptococcal infections, while substantiating their traditional Native American uses.

## Highlights

• *H. virginiana* extracts inhibited the growth of common bacterial skin pathogens.
• MIC values were determined and indicated strong inhibitory activity.
• The aqueous extract potentiated the antibacterial activity of chloramphenicol.
• The methanolic extracts potentiated activity of ciprofloxacin.
• All extracts were determined to be non-toxic by *Artemia* nauplii assays.

## Graphical abstract

**Figure:**
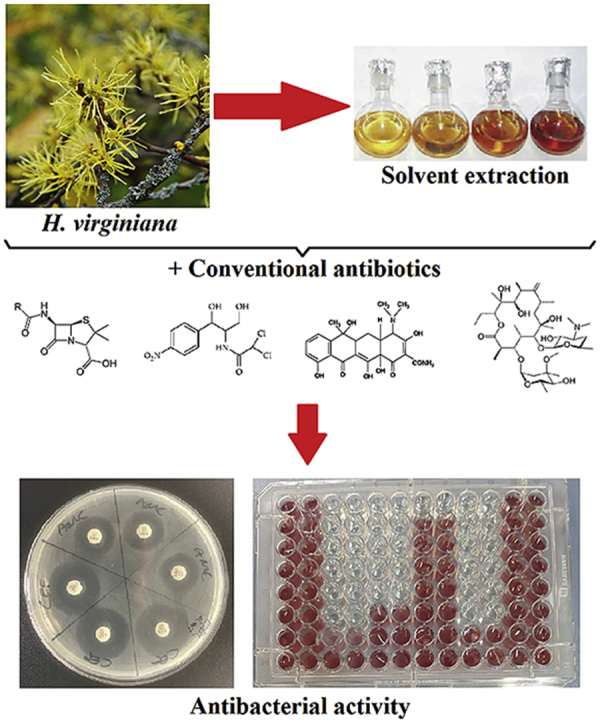

## Abbreviations

ALA: brine-shrimp lethality assay
DMSO: dimethyl sulfoxide
ΣFIC: the sum of the fractional inhibitory concentration
HAMA: hamamelitannin
INT: ρ-iodonitrotetrazolium chloride
LD_50_: dose of sample necessary which causes death of 50% of test organisms or cells
MH: Mueller Hinton
MIC: minimum inhibitory concentration
WH: witch hazel
ZOI: zone of inhibition

## 1 Introduction

*Hamamelis virginiana* L., commonly known as Virginian witch hazel (WH), is native to North America and has been utilised traditionally as a medicinal agent by indigenous Native American populations. Its primary applications are for the treatment of haemorrhoids, superficial skin wounds and skin inflammation. Decoctions of WH were prepared by boiling the shrub (leaves and/or bark) and applying it to the skin to reduce inflammation,^1^ although preparations were also ingested for colds and fevers.^2^

A number of studies have reported anti-inflammatory, antioxidant and anti-proliferative properties of WH extracts.^3^, ^4^, ^5^, ^6^, ^7^, ^8^ Its uses as an astringent and for the treatment of acne and irritable scalp conditions have also been documented.^9^, ^10^, ^11^, ^12^ Antiviral activities have also been reported.^13^^,^^14^ However, there is scant scientific evidence on the antibacterial effects of WH extracts. A 2002 study^15^ revealed weak activity of a WH distillate against the *Staphylococcus* species *S. aureus* and *S. epidermidis*, although the product contained 5% urea, which may have contributed to this activity.^16^ More recently, strong activities towards these two strains were demonstrated using a commercial WH product available from StaphOff Biotech Inc. (Hopkinton, USA) named whISOBAX.^17^ However, activities were measured based on phenolic content of the product rather than using crude extracts and therefore may not necessarily validate the traditional usage of this species. It should also be noted that this WH product is supplied as a tincture, and thus high concentrations of ethanol are present which would contribute substantially to the observed antibacterial activity. Polyphenolic compounds are well-known as bacterial growth inhibitors,^18^ although the predominant phenolic compound in WH, hamamelitannin (HAMA), does not appear to contribute to the antibacterial activity *in vitro*^17^^,^^19^ despite being capable of inhibiting staphylococcal infections *in vivo*.^20^ Since staphylococcal species are found predominantly on epithelial surfaces, any antibacterial properties of WH may account for some of the soothing, anti-inflammatory effects of the topical WH applications via beneficial anti-infective measures that reduce skin inflammation.

WH herbal tea is also taken orally to treat inflammation of the gums and mouth and as a mouthwash/gargle to treat inflammation of the oropharynx.^21^^,^^22^ Numerous streptococcal species are known to cause dental decay, including *S. mutans* and *S. oralis*, while *S. pyogenes* is a major cause of bacterial throat infections commonly known as “strep throat”. A commercial preparation of WH has been shown to decrease tooth biofilm formation,^23^ whilst a report from Korean researchers indicated that *S. mutans* growth was inhibited by Dickinson’s® Witch Hazel (T.N. Dickinson Co. USA), a commercial WH product.^24^ However, this product contains 14% ethanol, which may have compromised the assessment of bacterial growth inhibition by any available WH phytochemicals and yielded false positive outcomes.

Together, these data demonstrate that limited scientific evidence to support the use of WH as anti-infective agents exists in dental care or as a gargle for sore throat, or for the treatment of skin infections. Thus, due to the lack of meaningful studies on the antibacterial effects of WH extracts, the present study investigated the ability of WH extracts (prepared using solvents of varying polarity) to inhibit the growth of three streptococcal and two staphylococcal species on agar, and then to determine minimum inhibitory concentration (MIC) values using broth dilution experiments. Given the potential confounding factors present in previous studies this study intends to clarify the direct activity of extracts that are devoid of organic solvents present in many commercial preparations which complicates the quantification of antibacterial activities. To achieve this, dried crude extracts resuspended in aqueous solution are utilised in this study. Extracts showing appreciable activity were then combined with various conventional antibiotics to determine whether there are any interactions between the extracts and antibiotics. The toxicities and phytochemical profiles of the WH extracts were also assessed. Additionally, the phytochemical profiles of the WH extracts were assessed, since numerous other studies have found that medicinal plant extracts rich in tannins, flavonoids, polyphenols, triterpenoids and cardiac glycosides have been found to be effective at inhibiting the growth of staphylococcal and streptococcal species.^25^, ^26^, ^27^, ^28^, ^29^ Finally, the WH extracts were then used in brine shrimp lethality bioassays to ascertain their cytotoxicity.

## 2 Materials and methods

### 2.1 Plant source and extraction

WH leaf material was originally sourced from the US and obtained from Noodles Emporium (Australia) and supplied as small (approx. 5 mm) leaf fragments. Voucher specimens (GU2018WHa) are stored at the School of Environment and Science, Griffith University, Australia. Individual 1 g masses of the leaf material were weighed into separate tubes and 50 ml of either sterile deionised water, methanol, ethyl acetate, hexane or chloroform were added. All organic solvents were obtained from Ajax Fine Chemicals (Wollongong, Australia) and were AR grade. The leaves were extracted in each solvent for 24 h at room temperature with constant agitation and subsequently filtered through Whatman No. 54 filter paper to remove particulate matter. Organic solvents were evaporated by air drying at 45 °C for 36–48 h in a chemical flow-hood, whereas aqueous extracts were freeze-dried by lyophilization at −80 °C in a VirTis sentry 2.0 Bench Top Lyophilizer (SP Scientific, USA) for up to 48 h. The extracts were dried to completion, as determined by no further decreases in mass with subsequent weighing. All dried extracts were weighed to determine extraction yield, resuspended in 10 ml of sterile deionised water (containing 1% DMSO) and subjected to mild sonication (20 s pulses at 1 kHz, with 30 s rest between pulses). Extracts were then sterilised by passing through 0.22 μm Millex-GS mixed cellulose ester membrane syringe filter units (Merck Pty. Ltd., Baywater, Australia) and stored at 4 °C in tightly capped polypropylene tubes until required.

### 2.2 Qualitative phytochemical studies

Plant extract alkaloids, cardiac glycosides, flavonoids, phenolic compounds, phytosterols, saponins, tannins and triterpenoids were assessed using standard phytochemical assays.^30^

### 2.3 Bacterial cultures

*Streptococcus mutans* and *Streptococcus oralis* (wild-type, clinical isolates), *Streptococcus pyogenes* (ATCC 12384), *Staphylococcus epidermidis* (ATCC 122292) and *Staphylococcus aureus* (ATCC 25923) strains were used in this study. All dehydrated culture media were purchased from Oxoid Ltd. (Scoresby, Australia). *S. mutans*, *S. oralis* and *S. pyogenes* were grown at 37 °C in microaerophilic (5% CO_2_) conditions on Mueller-Hinton (MH) agar supplemented with 5% defibrinated horse blood for disc diffusion assays, and in brain heart infusion broth for liquid dilution assays. *S. epidermidis* and *S. aureus* were cultured aerobically at 37 °C on MH agar or MH broth containing 2% NaCl for the relevant assays. Streaked agar plates were cultured in parallel on general purpose, selective, and differential agar to ensure purity and for the phenotypic verification of the bacterial species used in all assays. The antibacterial test conditions conformed to CLSI standardised methods.^31^

### 2.4 Bacterial susceptibility to growth inhibition on agar

An assessment of bacterial susceptibility to inhibition by plant extracts or antibiotics on agar was conducted using a modified disc diffusion assay to confirm susceptibility and resistance.^32^^,^^33^ Antibiotics (Sigma-Aldrich Ltd., Australia) were included as controls against each bacterium: 10 μL of penicillin, erythromycin, tetracycline, chloramphenicol and ciprofloxacin solutions (containing 1 μg of each antibiotic) assayed, alongside control disks infused with 10 μL of deionised water. All extracts and antibiotics were tested in triplicate for each bacterial strain. One-way analysis of variance (ANOVA analysis was used to calculate statistical significance between control and treatment groups, or between treatment groups.

### 2.5 Liquid dilution MIC assay

A standard liquid dilution MIC assay^33^^,^^34^ was used to quantify bacterial growth inhibitory activity of the extracts and conventional antibiotics. Following an overnight incubation, 40 μL of 0.4 mg/ml ρ-iodonitrotetrazolium violet (Sigma-Aldrich Ltd., Australia) was added to each well and incubated for a further 4–6 h period at 37 °C to allow for colour development, where a pink-red colour indicated bacterial growth. The MIC was visually determined as the lowest dose at which colour development was inhibited. The reliability of MIC values was ensured by repeating the 96-well microtitre plate liquid dilution assays twice on separate days, with two replicates per assay, to confirm that the results were reproducible for all extracts and antibiotics tested.

### 2.6 Fractional inhibitory concentration (FIC) assessment

Interactions between the conventional antibiotics and WH extracts were examined by determination of the sum of fractional inhibitory concentrations (∑FIC) for each combination.^35^ In order to conduct these experiments, only WH extracts that possessed appreciable activities (<2000 μg/ml) were tested with antibiotics whose MIC values could be determined for each of the bacterial strains. Although MIC values < 1000 μg/ml are generally considered to be noteworthy,^36^ we decided to include extracts showing MIC values that were two-fold higher in order to provide a more expansive investigation of extract-antibiotic combinations. The FIC values for each component (a and b) were calculated using the following equations where a represents the plant extract sample and b represents the conventional antibiotic:

FIC (a) = MIC (a in combination with b) / MIC (a independently)

FIC (b) = MIC (b in combination with a) / MIC (b independently)

The ΣFIC was then calculated using the formula ΣFIC = FIC(a) + FIC(b). The interactions were classified as synergistic (ΣFIC ≤0.5), additive (ΣFIC >0.5–1.0), indifferent (ΣFIC >1.0–4.0) or antagonistic (ΣFIC >4.0).^37^

### 2.7 *Artemia franciscana* Kellogg nauplii toxicity screening

A modified *Artemia franciscana* nauplii lethality assay^38^ was used to assess the toxicity of the WH extracts. Dried *A. franciscana* eggs (Ocean Nutrition, CA, USA) were grown in Tropic Marine Salt artificial seawater for use in the assay. Potassium dichromate (AR grade, ChemSupply Pty. Ltd., Gillman, Australia) was used as a reference toxin and artificial seawater (Sigma-Aldrich Ltd., Australia) as a negative control. Extracts were tested at concentrations up to 1000 μg/ml. The LC_50_ values for each treatment was calculated using Probit analysis.

## 3 Results

### 3.1 Extraction yields and phytochemical screening

Yields produced from the extraction of 1 g of plant material were highest in the methanolic extracts (26.8 mg). Aqueous and chloroform extracts produced lower yields (19.7 and 16.4 mg, respectively), whilst the yields were relatively poor when hexane (8.2 mg) and ethyl acetate (5.1 mg) were used as the extracting solvents. The dried extractants were resuspended in deionised water (containing 1% DMSO) to produce the extract concentrations shown in Table 1. A series of qualitative phytochemicals tests were conducted on each extract. Phytochemicals were absent or below the level of detection in the hexane and chloroform extracts. The aqueous and methanolic extracts were rich in phenolics, flavonoids and tannins, whilst the ethyl acetate extract contained minor quantities of these phytochemicals. Cardiac glycosides and saponins were present in moderate abundances in the methanolic extract, and cardiac glycosides were also detected (in minor amounts) in the aqueous extract. The methanolic extract, but not the aqueous extract, contained triterpenoids. Phytosterols, alkaloids and anthraquinones were not detected in either of these extract preparations.

**Table 1:** Mass of dried extracted material, concentration after resuspension in 1% DMSO, and qualitative phytochemical screenings of the WH extracts used in the present study.

Extract	Mass of dried extract (mg)	Resuspended extract (mg/mL)	Phytochemical tested										
			Total phenolics	Water soluble phenols	Water insoluble phenols	Cardiac glycosides	Saponins	Triterpenoids	Phytosterols	Alkaloids	Flavonoids	Tannins	Anthraquinones
WH-Aq	197	19.7	+++	+++	+++	+	–	–	–	–	+++	+++	–
WH-MeOH	268	26.8	+++	+++	+++	++	++	+	–	–	+++	+++	–
WH-EtAc	51	5.1	+	+	+	–	–	–	–	–	+	+	–
WH-Hex	82	8.2	–	–	–	–	–	–	–	–	–	–	–
WH-CL	164	16.4	–	–	–	–	–	–	–	–	–	–	–

WH = witch hazel; Aq = aqueous; MeOH = methanol; EtAc = ethyl acetate; Hex = hexane; CL = chloroform. +++ indicates a large response in the assay; ++ indicates a moderate response; + indicates a minor response; - indicates no response.

### 3.2 Antibacterial activity on agar

**Fig. 1:**
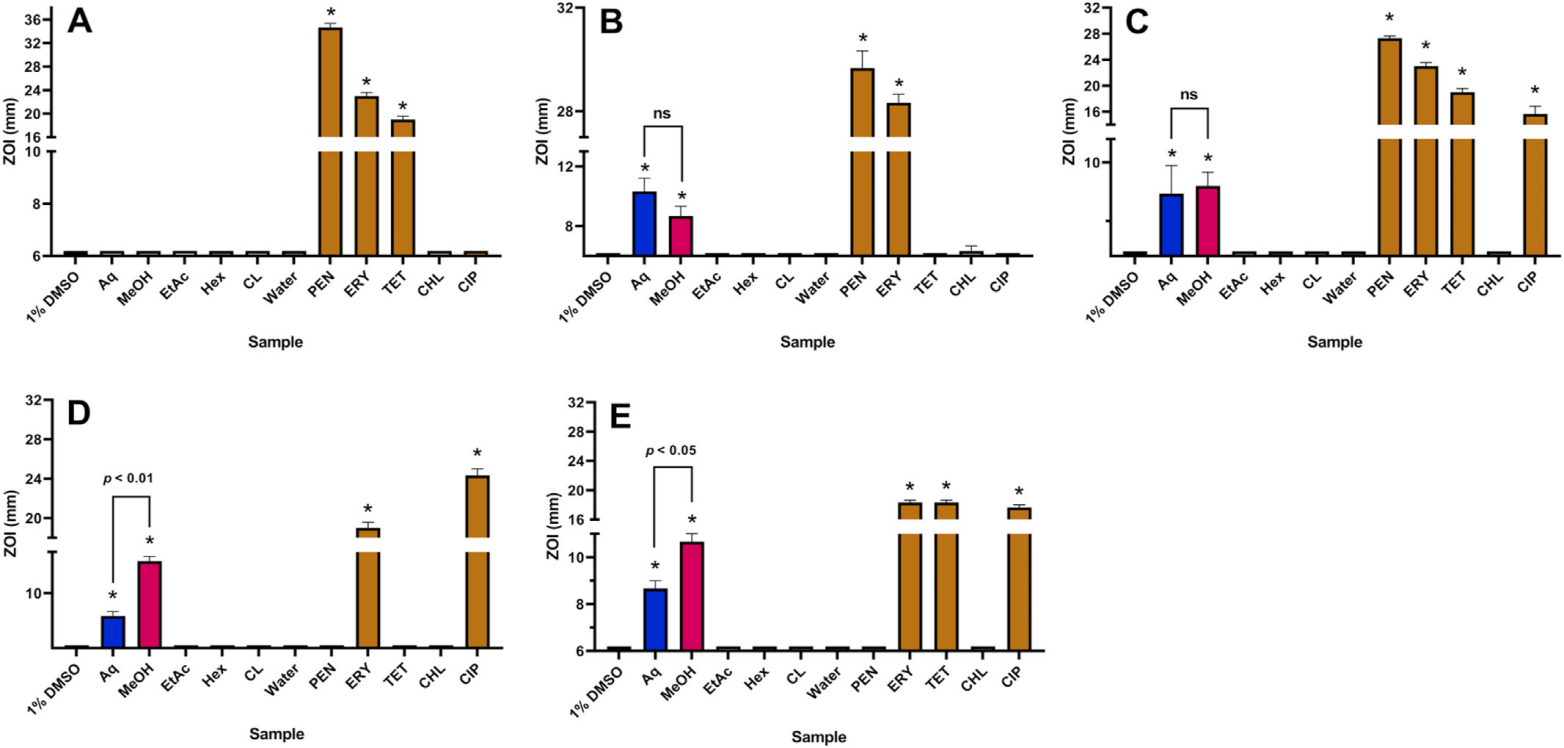
Antibacterial activity of WH extracts and reference antibiotics on agar against *S. mutans* (A), *S. oralis* (B), *S. pyogenes* (C), *S. epidermidis* (D) and *S. aureus* (E), measured as ZOI (mm). For the extract samples (10 μL per disc), Aq = aqueous; MeOH = methanolic; EtAc = ethyl acetate; Hex = hexane; CL = chloroform. Antibiotics (1 μg per disc) are PEN = penicillin; ERY = erythromycin; TET = tetracycline; CHL = chloramphenicol and CIP = ciprofloxacin. Results are expressed as mean zones of inhibition of triplicate assays ± SEM. Asterisk indicates results that are significantly different to the relevant negative control (*p* < 0.01); ns = not significant.

A series of disc diffusion assays were used to obtain a semi-quantitative assessment of the inhibition of bacterial growth on agar plates using 10 μL of the crude extracts that were resuspended in 1% DMSO, and compared to 1 μg of each of five conventional antibiotics (penicillin, erythromycin, tetracycline, chloramphenicol and ciprofloxacin). *S. mutans* was not susceptible to any of the extracts tested, whilst large ZOIs were observed for three of the reference antibiotics (Fig. 1A). In contrast, *S. oralis* and *S. pyogenes* were inhibited by both the aqueous and methanolic WH extracts (Fig. 1B and C), with ZOIs ranging from 8 to 11 mm. There were no significant differences in the magnitudes of inhibition between these two extracts against these two strains.

The two staphylococcal strains were also inhibited by the aqueous and methanolic WH extracts, although in these cases the methanolic extract produced a significantly greater inhibition of *S. epidermidis* (Fig. 1D; *p* < 0.01) and *S. aureus* (Fig. 1E; *p* < 0.05) than the aqueous extract. ZOIs of up to 13 mm for *S. epidermidis* were observed for the methanolic extract. Contrastingly, ZOIs were not observed for the ethyl acetate, hexane and chloroform extracts against any of the bacterial species.

### 3.3 MIC quantification

Further analysis of plant extract activity was determined using both undiluted crude extracts as well as extracts diluted with broth, in order to more precisely determine MIC values of each extract against the five bacterial strains used in this study. The MIC values of the extracts are shown in Table 2, alongside the MIC values of the reference antibiotic controls. Initially, two general trends were deduced from these results. The ethyl acetate, hexane and chloroform WH extracts did not inhibit the growth of any of the bacterial strains at the highest concentrations of extracts tested and were thus deemed inactive against the streptococcal and staphylococcal species used in this study. Furthermore, the activities of the control antibiotics in the liquid dilution assays (Table 2) were generally concordant with the disc diffusion inhibitory activities on agar (Fig. 1). All bacterial strains demonstrated some form of resistance to antibacterial controls with MICs >1 μg/ml shown for at least one agent (Table 2).

**Table 2:** MIC values (μg/mL) for WH extracted with water (Aq), methanol (MeOH), ethyl acetate (EtAc), hexane (Hex) and chloroform (CL) and for the conventional antibiotics PEN (penicillin), ERY (erythromycin), TET (tetracycline), CHL (chloramphenicol) and CIP (ciprofloxacin) against the five bacterial strains tested in this study.

Extract or antibiotic	MIC (μg/mL)				
	S. mutans	S. oralis	S. pyogenes	S. epidermidis	S. aureus
WH-Aq	4925	1478	1724	308	493
WH-MeOH	>10000	838	503	210	251
WH-EtAc	>10000	>10000	>10000	>10000	>10000
WH-Hex	>10000	>10000	>10000	>10000	>10000
WH-CL	>10000	>10000	>10000	>10000	>10000
PEN	<0.02	0.078	<0.02	>2.5	>2.5
ERY	0.039	0.039	0.039	0.156	0.313
TET	0.625	>2.5	0.078	>2.5	0.156
CHL	2.5	2.5	2.5	2.5	>2.5
CIP	2.5	2.5	0.625	0.156	0.625

WH = witch hazel. Values > 2.5 indicate lack of growth inhibition at the highest concentration of antibiotic examined. The range of concentrations used in the assays was 0.01–10 mg/ml for the plant extracts and 0.01–2.5 μg/ml for the reference antibiotics.

The methanolic extract was inactive against *S. mutans* while the aqueous extract possessed a very low activity (MIC = 4925 μg/ml) against this strain, suggesting that *S. mutans* is almost completely resistant to the WH extracts. Contrastingly, the other 4 strains were susceptible to the aqueous and methanolic WH extracts, with the methanolic extract being generally more potent than the aqueous counterpart (Table 2). Moderate activity was observed against *S. oralis* for the aqueous extracts (MIC = 1478 μg/ml) and noteworthy activity for the methanolic extract (MIC = 838 μg/ml). Similar findings for these extracts were observed for *S. pyogenes*. However, these extracts were much more powerful inhibitors of staphylococcal growth, particularly the methanolic extract, which produced low MIC values for *S. epidermidis* (308 μg/ml) and *S. aureus* (251 μg/ml) with a low MIC value also calculated for the aqueous extract against *S. epidermidis* (308 μg/ml). It should also be noted that these extracts produced similar growth inhibition profiles on agar (Fig. 1D and E), with the methanol extract being more potent than the water extract in each case. This suggests that WH preparations extracted with methanol or water are effective inhibitors of the growth of these two staphylococcal strains in both semi-solid and liquid culture environments (approximating epidermal and soft tissue infections, as well as gastric and blood infections).

### 3.4 FIC determinations

FIC values were acquired using 1:1 ratios of the aqueous or methanolic WH extracts to conventional antibiotics. *S. mutans* was not included in these assays as this strain was not inhibited by any of the extracts. The sum of FIC (∑FIC) could be calculated for the other four strains using these extracts but only in cases where they were combined with antibiotics that produced an MIC (see Table 2) for the strains. Antibiotics that were unable to inhibit the growth of the bacterial strain, or inhibited the strain at the lowest concentration tested, did not allow for the calculation of ∑FIC in such cases.

Table 3 shows the FIC values for the extracts and the antibiotic which contribute to the ∑FIC value in each case. There were only two instances where additive interactions were found between extract and an antibiotic. Specifically, these were aqueous WH extract combined with chloramphenicol against *S. oralis*, and methanolic WH extract combined with ciprofloxacin against *S. aureus* (∑FIC = 0.75 in both cases). This indicates that the use of both components to treat infections caused by these pathogens may hasten clearance of the infection. Although the remaining combinations that were tested were non-interactive, it is noteworthy that antagonistic interactions were not observed, suggesting that combinations of the extracts and antibiotics do not reduce the antimicrobial effects of each component in these cases and thus would not hamper the therapeutic outcomes should both agents be used concomitantly at the site of infection.

**Table 3:**
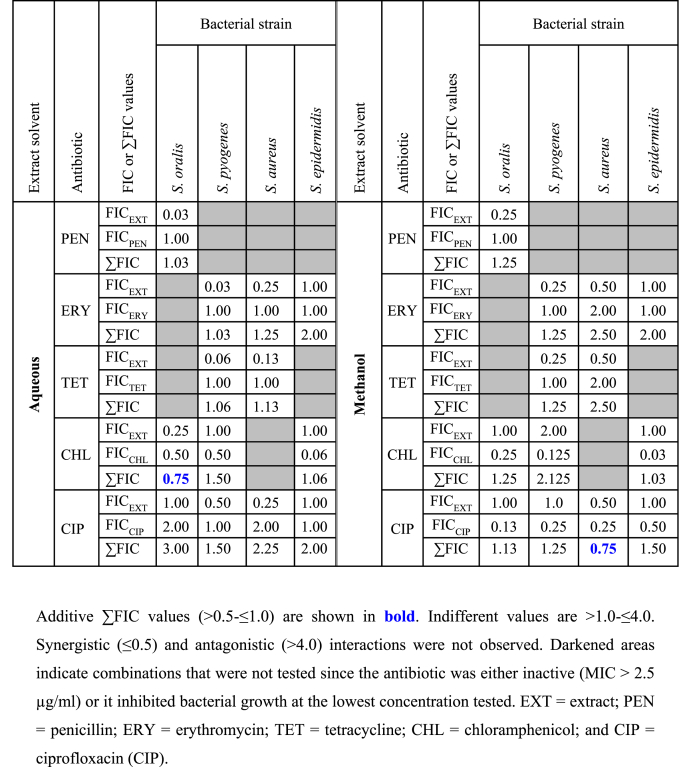
FIC and ∑FIC values, where relevant, for the combinations of the aqueous (left) and methanolic (right) WH extracts with antibiotics against the bacterial species used in this study.

### 3.5 Toxicity quantification

Each of the WH extracts were diluted in artificial seawater across a range of concentrations for testing in the *Artemia* nauplii bioassay. The % mortality for all extracts was not significantly different to that of the negative control (0%). LC_50_ values could not be determined for any of the extracts, as less than 50% mortality was observed for all concentrations examined following 24 h exposure, including at 1000 μg/mL. As such, all extracts were deemed non-toxic.^39^

## 4 Discussion

Interest in the usage of traditional medicines as anti-infective therapies has increased in recent years, particularly against resistant bacterial strains (as reviewed by^40^). It is commonly believed (often erroneously) that plant derived antibacterial therapies are safer and have lower toxicity than conventional antibiotics, yet may possess similar efficacy. Indeed, numerous studies published in recent years have reported potent antibacterial activity for plant extracts^34^^,^^41^^,^^42^ and essential oils,^43^^,^^44^ even when tested against multiple antibiotic-resistant bacterial strains.^45^ In this study, we sought to test *H. virginiana* extracts for growth inhibitory activity against a panel of *Staphylococcus* and *Streptococcus* species. These bacteria were selected for study as these genera consist of some of the most important bacterial pathogens for humans. *Staphylococcus* and *Streptococcus* spp. bacteria are extremely versatile and can infect a wide variety of tissues, causing multiple diseases, dependent on the tissue infected. For example, *S. aureus* is a common cause of boils, folliculitis, impetigo and cellulitis when it infects skin and soft tissue.^46^ The same bacterium can also cause gastrointestinal distress and is a common cause of food poisoning. In bone and joints (mainly following surgery), *S. aureus* infections may manifest as osteomyelitis or septic arthritis. Furthermore, when blood infections or infections of cardiac tissue occur, *S. aureus* infections may also cause bacteraemia and infective endocarditis respectively. *Streptococcus* spp. are similarly versatile. For example, *S. pyogenes* is a common cause of bacterial pharyngitis when it infects the throat, impetigo and cellulitis when it infects the skin and food poisoning in the gastrointestinal system.^47^ It may also cause a suite of autoimmune conditions including rheumatic fever, rheumatic heart disease and scarlet fever in genetically susceptible individuals.^48^ Of further concern, many *Staphylococcus* and *Streptococcus* strains have developed extensive antibiotic resistance to all major classes of antibiotics^40^ and the development of effective new therapies against these pathogens is urgently required.

*H. virginiana* is best known as a treatment for epidermal conditions, including superficial skin wounds, skin inflammation and haemorrhoids. Surprisingly, there is a lack of studies examining the antibacterial properties of this plant, despite studies reporting high levels of tannins in *H. virginiana* extracts.^7^ Our study screened *H. virginiana* extracts of varying polarity and found appreciable inhibitory activities for the higher polarity methanolic and aqueous extracts against most *Staphylococcus* and *Streptococcus* spp. Only *S. mutans* remained relatively unaffected by these extracts. The methanolic extract was a particularly good inhibitor of the other tested bacteria, with MIC values between 210 and 840 μg/ml. The potency of these extracts is particularly promising as all of the tested bacteria displayed resistance (MICs >1 μg/ml) towards conventional antibiotics. Resistance patterns were also similar to those found in isolates from clinical practice reflecting potential for study results to be applicable to clinical infectious disease.^49^ Chloramphenicol resistance was particularly widespread, with all bacterial species tested displaying resistance to that antibiotic. Penicillin was similarly ineffective, with only *S. oralis* being sensitive to its actions. The most effective of the antibiotics was erythromycin, with all *Staphylococcus* and *Streptococcus* spp. being susceptible (MIC values generally 0.04–0.3 μg/ml). The considerable activity of the methanolic and aqueous *H. virginiana* extracts against these multi-antibiotic resistant species indicates their potential in the development of novel antibiotic therapies. Growth inhibitory activity against these bacteria also indicates that the extracts may function via mechanisms which differ from those of the conventional antibiotics tested in this study. Although this study did not report synergistic relationships between WH and the conventional antibiotics tested, exploration of an extended range of antibiotic combinations is warranted to determine if the extracts may contain resistance-modifying agents that inhibit bacterial resistance mechanisms, allowing other compounds to function with greater potency. Further studies targeting known resistance mechanisms may also be useful in determining if WH possesses a novel mechanism of antibacterial action or if it is capable of targeting resistance mechanisms. This may have far greater implications than developing a new pharmacophore for potential drug development. Indeed, if the extracts do inhibit bacterial resistance mechanisms, they may allow clinical antibiotics to function again against bacteria that are otherwise resistant to their actions.

Surprisingly few plant-derived antibiotic therapies are currently used clinically, possibly due to the pharmaceutical industry’s preference for monotherapies over multiple component formulations.^50^ However, the therapeutic potency of crude plant preparations is often lost or significantly decreased when individual fractionated components are used in isolation. Single molecular components often require ancillary constituents to potentiate their activity or to block resistance mechanisms that render bacteria refractory to their actions. Thus, multi-component combinational systems have attracted recent attention and an increasing number of recent studies have focussed on combinations of plant preparations (or isolated components),^43^^,^^44^ as well as combinations containing plant components and conventional antibiotics.^35^^,^^51^, ^52^, ^53^, ^54^, ^55^ Interestingly, many plant preparations are synergistic potentiators of conventional antibiotics, even when the plant preparations do not themselves have antibacterial activity.^56^ For this reason, a major focus of our study was to determine the interactive effects of the *H. virginiana* extracts when tested in combination with selected conventional antibiotics.

We were unable to obtain ΣFIC values and determine the class of interaction for many of the combinations tested in this study as one or both components were completely ineffective in the growth inhibitory assays. This was particularly evident for penicillin. Since only *S. oralis* was susceptible to this antibiotic when tested alone, we could not determine ΣFIC values for any penicillin containing combination against any of the other bacteria. Of the combinations for which we were able to calculate ΣFIC values, none displayed either synergistic or antagonistic combinational effects. The majority demonstrated non-interactive combinational profiles. Whilst these combinations would have no added benefit over the individual monotherapies, the use of the plant preparation concurrently with the conventional antibiotics would not further decrease the efficacy of the antibiotic component. Notably, two additive interactions were also observed (aqueous extract in combination with chloramphenicol against *S. oralis*; and methanolic extract in combination with ciprofloxacin against *S. aureus*). Whilst these effects are less pronounced than synergistic interactions, additive interactions also result in enhanced efficacy, thereby allowing lower doses to be administered, thus reducing any side effects of the chemotherapy.^40^ The exposure of bacteria to lower levels of antibiotics may also decrease the induction of further antibiotic resistance in those bacteria. Further studies are required to examine these resistance mechanisms to better understand the mechanisms and thereby tailor combinations to different diseases. Future studies expanding the range of antibacterial drugs tested and the combinations of these agents will further increase knowledge in this area.

Studies such as this are not only important to identify potentiating combinations which overcome bacterial antibiotic resistance. They may also provide valuable information to inform on combinational drug usage in a clinical setting where interactions between herbal/traditional medicines and conventional therapies are commonplace. Patients may use both traditional and conventional therapeutics concurrently without understanding the potential for drug interactions, and often without informing their medical practitioner. Mixing therapy modalities may impact on the efficacy of both therapies and may pose serious risks to patient safety.^57^^,^^58^ Of concern, the practice of combining herbal and conventional medicines is prevalent, even in Western countries where allopathic medicine dominates. Indeed, a survey conducted in the United States of America reported that up to 84% of patients regularly use allopathic medicines concurrently with natural therapies, often believing that combining the two treatment methods would result in enhanced effects.^58^ However, combinations of plant products and conventional medicines often have decreased efficacy or can result in severe reactions. Some of these cross-reactive and counter-indicative combinations have been documented,^59^ yet the interactive effects of most combinations are yet to be investigated. Using a brine shrimp toxicity model, the WH extracts prepared in our study were non-toxic. Studies in mice have indicated that ethanolic WH extracts do not possess toxicity *in vivo* at systemic concentrations up to 300 mg/kg.^60^, ^61^ Furthermore, studies on human dermal fibroblast cells have demonstrated a protective role of WH extracts against H_2_O_2_-induced damage, possibly due to their free radical scavenging capabilities.^7^ Together, this highlights the potential for the safe use of WH extracts as a therapeutic option for the treatment of both systemic and superficial staphylococcal and streptococcal infections.

The active extracts in this study were found to possess considerable levels of tannins and flavonoids with moderate levels of cardiac glycosides, saponins and triterpenoids present in the methanolic extract. Plant-derived tannins are known to possess antibacterial activity, including against *S. aureus*^41^^,^^62^^,^^63^ and *S. pyogenes*.^64^ WH has been reported to contain 3–10% tannin content, however the HAMA tannin found in this plant does not possess activity in isolation,^17^^,^^19^ suggesting that other tannins may either be responsible or are required in combination with HAMA for antibacterial effects. WH is known to also contain a variety of different tannin compounds^14^ which may have roles in bacterial growth inhibition. Flavonoids are also strong antibacterial compounds,^65^ and it is noteworthy that the active extracts in the present study contained high levels of flavonoids. However, the identity of any individual flavonoid molecules that may be active against streptococcal and staphylococcal species in the present study remains unknown. Further investigations into the molecular compounds, or combination of compounds, present in the active WH extracts will be carried out in order to identify potential drug targets that may treat infections triggered by streptococcal and staphylococcal bacteria.

In contrast with the active extracts, only minor quantities of tannins and flavonoids were present in the ethyl acetate extract, while these phytochemicals could not be detected in the hexane and chloroform extracts. Since the ethyl acetate, hexane and chloroform extracts also lacked antibacterial activity, it is apparent that the low abundance (or absence) of phytochemicals in these extracts are responsible for their observed lack of activity. However, the identity of the specific class or classes of phytochemicals responsible for the observed activity cannot be conclusively determined here. Evidence from numerous studies have revealed potential mechanisms of action for the types of compounds from phytochemical classes detected in active extracts in our study. For example, tannins extracted from other medicinal plants appears to elicit multiple effects on staphylococcus by affecting osmotic and pH regulation, metabolic pathways, and via inhibition of the catalase enzyme as antimicrobial mechanisms.^65^^,^^66^ Flavonoids are postulated to attenuate bacterial growth and pathogenicity by altering cytoplasmic membrane function and permeability, and inhibiting DNA gyrase.^67^, ^68^, ^69^ The bacterial cell surface may also be the target of plant-derived polyphenols^70^ by inducing permeabilization,^71^ while the triterpenoids (saponins) appear to act as anti-staphylococcal agents in similar fashion, in addition to being capable of halting protein synthesis and reducing biofilm formation.^72^^,^^73^

## 5 Conclusions

WH has been used for thousands of years to treat numerous ailments associated with epithelial surfaces. Our study appears to be the first report of the antibacterial properties of WH plant leaves extracted with polar solvents (water and methanol) with the extracted phytochemicals being resuspended in an aqueous solution devoid of urea, ethanol or other contaminating organic solvents that are often found in commercial WH products. Significant activities were observed against streptococcal and staphylococcal species of major medical importance. These strains are versatile in the types of infections they trigger and are also renowned for their propensity for resistance. Although a case could be made for the phenolic, triterpenoid and tannin compounds in the active WH extracts being responsible for activity, further study is necessary in order to determine the molecular constituent(s) that contribute to the observed antibacterial effects, and/or whether defined combinations of these phytochemicals are necessary for activity. Combinations with the antibiotics tested in this study does not enhance activities in most cases, suggesting that the focus of future studies should be directed at isolating the WH phytochemicals that inhibit bacterial growth. Such compounds may prove to be effective novel antibiotics with clinical applications in the treatment of streptococcal and staphylococcal infections, including the highly resistant strains which have emerged in recent decades.

## Source of support

Financial support for this work was provided by the Environmental Futures Research Institute and the Quality Use of Medicines (QUM) Network, 10.13039/501100001791Griffith University, Australia.

## Taxonomy classification

Control of skin disease through the eradication of bacterial skin pathogens.

## Declaration of competing interest

The authors declare that they have no competing interests.

## References

[1] Korting H.C., Schafer-Korting M., Hart H., Laux P., Schmid M. (1993). Anti-inflammatory activity of hamamelis distillate applied topically to the skin. Eur J Clin Pharmacol.

[2] Moerman D.E. (1986).

[3] Swoboda M., Meurer J. (1991). Treatment of atopic dermatitis with hamamelis ointment. Br J Phytother.

[4] Duwiejua M., Zeitlin I.J., Waterman P.G., Gray A.I. (1994). Anti-inflammatory activity of *Polygonum bistorta, Guaiacum officinale* and *Hamamelis virginiana* in rats. J Pharm Pharmacol.

[5] Hughes-Formella B.J., Bohnsack K., Rippke F. (1998). Anti-inflammatory effect of hamamelis lotion in a UVB erythema test. Dermatology.

[6] Deters A., Dauer A., Schnetz E., Fartasch M., Hensel A. (2001). High molecular compounds (polysaccharides and proanthocyanidins) from *Hamamelis virginiana* bark: influence on human skin keratinocyte proliferation and differentiation and influence on irritated skin. Phytochemistry.

[7] Tourino S., Lizarraga D., Carreras A. (2008). Highly galloylated tannin fractions from witch hazel (*Hamamelis virginiana*) bark: electron transfer capacity, *in vitro* antioxidant activity, and effects on skin-related cells. Chem Res Toxicol.

[8] Sánchez-Tena S., Fernandez-Cachon M.L., Carreras A. (2012). Hamamelitannin from witch hazel (*Hamamelis virginiana*) displays specific cytotoxic activity against colon cancer cells. J Nat Prod.

[9] Odukoya O.A., Sofidiya M.O., Ilori O.O. (2009). Hemorrhoid therapy with medicinal plants: astringency and inhibition of lipid peroxidation as key factors. Int J Biol Chem.

[10] Reuter J., Wölfle U., Weckesser S., Schempp C. (2010). Which plant for which skin disease? Part 1: atopic dermatitis, psoriasis, acne, condyloma and herpes simplex. J Dtsch Dermatol Ges.

[11] Chularojanamontri L., Tuchinda P., Kulthanan K., Pongparit K. (2014). Moisturizers for acne: what are their constituents?. J Clin Aesthet Dermatol.

[12] Trüeb R.M. (2014). North American Virginian witch hazel (*Hamamelis virginiana*): based scalp care and protection for sensitive scalp, red scalp, and scalp burn-out. Int J Trichol.

[13] Erdelmeier C.A., Cinatl J., Rabenau H., Doerr H.W., Biber A., Koch E. (1996). Antiviral and antiphlogistic activities of *Hamamelis virginiana* bark. Planta Med.

[14] Theisen L.L., Erdelmeier C.A., Spoden G.A. (2014). Tannins from *Hamamelis virginiana* bark extract: characterization and improvement of the antiviral efficacy against influenza A virus and human papillomavirus. PloS One.

[15] Gloor M., Reichling J., Wasik B., Holzgang H.E. (2002). Antiseptic effect of a topical dermatological formulation that contains Hamamelis distillate and urea. Complement Med Res.

[16] Patil M., Poyil A.N., Joshi S.D., Patil S.A., Patil S.A., Bugarin A. (2019). Synthesis, molecular docking studies, and antimicrobial evaluation of new structurally diverse ureas. Bioorg Chem.

[17] Rasooly R., Molnar A., Choi H.Y., Do P., Racicot K., Apostolidis E. (2019). *In-vitro* inhibition of staphylococcal pathogenesis by witch-hazel and green tea extracts. Antibiotics.

[18] Bouarab-Chibane L., Forquet V., Lantéri P. (2019). Antibacterial properties of polyphenols: characterization and QSAR (quantitative structure–activity relationship) models. Front Microbiol.

[19] Leong C., Schmid B., Buttafuoco A., Glatz M., Bosshard P.P. (2019). *In vitro* efficacy of antifungal agents alone and in shampoo formulation against dandruff-associated *Malassezia* spp. and *Staphylococcus* spp. Int J Cosmet Sci.

[20] Kiran M.D., Giacometti A., Cirioni O., Balaban N. (2008). Suppression of biofilm related, device-associated infections by staphylococcal quorum sensing inhibitors. Int J Artif Organs.

[21] European Medicines Agency Committee on Herbal Medicinal Products (HMPC) (2020). Assessment report on *Hamamelis virginiana* L., cortex; Hamamelis virginiana L., folium; *Hamamelis virginiana* L., folium et cortex aut ramunculus destillatum, EMA/HMPC/114585/2008. http://www.ema.europa.eu/docs/en_GB/document_library/Herbal_-_HMPC_assessment_report/2010/04/WC500089242.pdf.

[22] Henley-Smith C.J., Botha F.S., Lall N., Méndez-Vilas A. (2013). *Microbial Pathogens and Strategies for Combating them: Science, Technology and Education.* Badajoz.

[23] Mouchrek J.C., Nunes L.H., Arruda C.S. (2015). Effectiveness of oral antiseptics on tooth biofilm: a study *in vivo*. J Contemp Dent Pract.

[24] Ryu S.Y., Ahn H.J., Kwon J.S., Park J.H., Kim J.Y., Choi J.H. (2008). The anti-bacterial effect of Witch Hazel (*Hamamelis virginiana*) on oral pathogens. J Oral Med Pain.

[25] Jamkhande P.G., Wattamwar A.S. (2015). *Annona reticulata* Linn. (Bullock’s heart): plant profile, phytochemistry and pharmacological properties. J Tradit Complement Med.

[26] Ahmed A.A., Sharmen F., Mannan A., Rahman M.A. (2015). Phytochemical, analgesic, antibacterial, and cytotoxic effects of *Alpinia nigra* (Gaertn.) Burtt leaf extract. J Tradit Complement Med.

[27] Mahomoodally M.F., Dilmohamed S. (2016). Antibacterial and antibiotic potentiating activity of *Vangueria madagascariensis* leaves and ripe fruit pericarp against human pathogenic clinical bacterial isolates. J Tradit Complement Med.

[28] Bhagavathy S., Mahendiran C., Kanchana R. (2019). Identification of glucosyl transferase inhibitors from *Psidium guajava* against *Streptococcus mutans* in dental caries. J Tradit Complement Med.

[29] Srifuengfung S., Bunyapraphatsara N., Satitpatipan V. (2020). Antibacterial oral sprays from Kaffir lime (*Citrus hystrix* DC.) fruit peel oil and leaf oil and their activities against respiratory tract pathogens. J Tradit Complement Med.

[30] Vesoul J., Cock I.E. (2011). An examination of the medicinal potential of *Pittosporum phylliraeoides*: toxicity, antibacterial and antifungal activities. Pharmacogn Commun.

[31] CLSI (2019).

[32] Cock I.E. (2018). Antimicrobial activity of *Aloe barbadensis* Miller leaf gel components. Internet J Microbiol.

[33] Tiwana G., Cock I.E., White A., Cheesman M.J. (2020). Use of specific combinations of the triphala plant component extracts to potentiate the inhibition of gastrointestinal bacterial growth. J Ethnopharmacol.

[34] Hübsch Z., van Zyl R.L., Cock I.E., van Vuuren S.F. (2014). Interactive antimicrobial and toxicity profiles of conventional antimicrobials with Southern African medicinal plants. South Afr J Bot.

[35] Ilanko A., Cock I.E. (2019). The interactive antimicrobial activity of conventional antibiotics and *Petalostigma* spp. extracts against bacterial triggers of some autoimmune inflammatory diseases. Pharmacogn J.

[36] Van Vuuren S.F. (2008). Antimicrobial activity of South African medicinal plants. J Ethnopharmacol.

[37] Doern C.D. (2014). When does 2 plus 2 equal 5? A review of antimicrobial synergy testing. J Clin Microbiol.

[38] Shalom J., Cock I.E. (2018). *Terminalia ferdinandiana* Exell. fruit and leaf extracts inhibit proliferation and induce apoptosis in selected human cancer cell lines. Nutr Canc.

[39] Ruebhart D.R., Wickramasinghe W., Cock I.E. (2009). Protective efficacy of the antioxidants vitamin E and Trolox against *Microcystis aeruginosa* and microcystin-LR in *Artemia franciscana* nauplii. J Toxicol Environ Health A..

[40] Cheesman M.J., Blonk B., Ilanko A., Cock I.E. (2017). Developing new antimicrobial therapies: are synergistic combinations of plant extracts/compounds with conventional antibiotics the solution?. Phcog Rev.

[41] Courtney R, Sirdaarta J, Matthews B, Cock IE. Tannin components and inhibitory activity of Kakadu plum leaf extracts against microbial triggers of autoimmune inflammatory diseases. Pharmacogn J 7:18–31. doi:10.5530/pj.2015.7.2.

[42] Winnett V., Sirdaarta J., White A., Clarke F.M., Cock I.E. (2017). Inhibition of *Klebsiella pneumoniae* growth by selected Australian plants: natural approaches for the prevention and management of ankylosing spondylitis. Inflammopharmacology.

[43] Van Vuuren S.F., Viljoen A.M. (2006). A comparative investigation of the antimicrobial properties of indigenous South African aromatic plants with popular commercially available essential oils. J Essent Oil Res.

[44] Suliman S., van Vuuren S.F., Viljoen A.M. (2010). Validating the *in vitro* antimicrobial activity of *Artemisia afra* in polyherbal combinations to treat respiratory infections. South Afr J Bot.

[45] Cheesman M.J., White A.R., Matthews B., Cock I.E. (2019). *Terminalia ferdinandiana* fruit and leaf extracts inhibit methicillin-resistant *Staphylococcus aureus* growth. Planta Med.

[46] Tong S.Y.C., Davis J.S., Eichenberger E., Holland T.L., Fowler V.G. (2015). *Staphylococcus aureus* infections: epidemiology, pathophysiology, clinical manifestations, and management. Clin Microbiol Rev.

[47] Efstratiou A., Lamagni T., Ferretti J.J., Stevens D.L., Fischetti V.A. (2016). Streptococcus Pyogenes: Basic Biology to Clinical Manifestations.

[48] Cock I.E., Cheesman M.J., Watson R., Preedy V. (2019). Bioactive Food as Dietary Interventions for Arthritis and Related Inflammatory Diseases..

[49] (2020). Australian Medicines Handbook.

[50] Cock I.E. (2018). Is the pharmaceutical industry’s preoccupation with the monotherapy drug model stifling the development of effective new drug therapies?. Inflammopharmacology.

[51] D’Arrigo M., Ginestra G., Mandalari G., Furneri P.M., Bisignano G. (2010). Synergism and postantibiotic effect of tobramycin and *Melaleuca alternifolia* (tea tree) oil against *Staphylococcus aureus* and Escherichia coli. Phytomedicine.

[52] Van Vuuren S.F., Viljoen A. (2011). Plant-based antimicrobial studies-methods and approaches to study the interaction between natural products. Planta Med.

[53] Hutchings A., Cock I.E. (2018). An interactive antimicrobial activity of *Embelica officinalis* Gaertn. Fruit extracts and conventional antibiotics against some bacterial triggers of autoimmune inflammatory diseases. Pharmacogn J.

[54] Mandeville A., Cock I.E. (2018). *Terminalia chebula* Retz. fruit extracts inhibit bacterial triggers of some autoimmune diseases and potentiate the activity of tetracycline. Indian J Microbiol.

[55] Ilanko P., McDonnell P.A., van Vuuren S., Cock I.E. (2019). Interactive antibacterial profile of *Moringa oleifera* Lam. extracts and conventional antibiotics against bacterial triggers of some autoimmune inflammatory diseases. South Afr J Bot.

[56] Aiyegoro O.A., Okoh A.I. (2009). Use of bioactive plant products in combination with standard antibiotics: implications in antimicrobial chemotherapy. J Med Plants Res.

[57] Butterweck V., Derendorf H. (2012). Herb-drug interactions. Planta Med.

[58] Vieira M., Huang S.-M. (2012). Botanical-drug interactions: a scientific perspective. Planta Med.

[59] Cock I.E. (2015). The safe usage of herbal medicines: counter-indications, cross-reactivity and toxicity. Pharmacogn Commun.

[60] Qinna N.A. (2013). Safety profile of suppository *Hamamelis virginiana* leaf extract. J Med Plants Res.

[61] Widsten P., Cruz C.D., Fletcher G.C., Pajak M.A., McGhie T.K. (2014). Tannins and extracts of fruit byproducts: antibacterial activity against foodborne bacteria and antioxidant capacity. J Agric Food Chem.

[62] Pereira A.V., Santana G.M., Góis M.B., Sant’Ana D.G., Méndez-Vilas A. (2015). The Battle against Microbial Pathogens: Basic Science, Technological Advances and Educational Programs.

[63] Hull S.V., Tucci M., Benghuzzi H. (2011). Evaluation of the antimicrobial efficacy of green tea extract (egcg) against *Streptococcus pyogenes* in vitro-biomed 2011. Biomed Sci Instrum.

[64] Farhadi F., Khameneh B., Iranshahi M., Iranshahy M. (2019). Antibacterial activity of flavonoids and their structure-activity relationship: an update review. Phytother Res.

[65] Carvalho R.S., Carollo C.A., de Magalhães J.C., Palumbo J.M.C., Boaretto A.G. (2018). e Sá IN, Ferraz AC, Lima WG, de Siqueira JM, Ferreira JMS. Antibacterial and antifungal activities of phenolic compound-enriched ethyl acetate fraction from *Cochlospermum regium* (mart. Et. Schr.) Pilger roots: mechanisms of action and synergism with tannin and gallic acid. South Afr J Bot.

[66] Liu M., Feng M., Yang K. (2020). Transcriptomic and metabolomic analyses reveal antibacterial mechanism of astringent persimmon tannin against Methicillin-resistant *Staphylococcus aureus* isolated from pork. Food Chem.

[67] Cushnie T.T., Lamb A.J. (2005). Antimicrobial activity of flavonoids. Int J Antimicrob Agents.

[68] Farhadi F., Khameneh B., Iranshahi M., Iranshahy M. (2019). Antibacterial activity of flavonoids and their structure–activity relationship: an update review. Phytother Res.

[69] Xie Y., Yang W., Tang F., Chen X. (2015). Ren, L. Antibacterial activities of flavonoids: structure-activity relationship and mechanism. Curr Med Chem.

[70] Bouarab-Chibane L., Forquet V., Lantéri P. (2019). Antibacterial properties of polyphenols: characterization and QSAR (Quantitative structure–activity relationship) models. Front Microbiol.

[71] Bhattacharya D., Ghosh D., Bhattacharya S. (2018). Antibacterial activity of polyphenolic fraction of Kombucha against *Vibrio cholerae*: targeting cell membrane. Lett Appl Microbiol.

[72] Chung P.Y. (2020). Novel targets of pentacyclic triterpenoids in *Staphylococcus aureus*: a systematic review. Phytomedicine.

[73] Dong S., Yang X., Zhao L., Zhang F., Hou Z., Xue P. (2020). Antibacterial activity and mechanism of action saponins from *Chenopodium quinoa* Willd. husks against foodborne pathogenic bacteria. Ind Crop Prod.

